# Microfluidic Monodispersed Microbubble Generation for Production of Cavitation Nuclei

**DOI:** 10.3390/mi15121531

**Published:** 2024-12-23

**Authors:** Renjie Ning, Blake Acree, Mengren Wu, Yuan Gao

**Affiliations:** Department of Mechanical Engineering, The University of Memphis, Memphis, TN 38152, USA; rning@memphis.edu (R.N.); bacree@memphis.edu (B.A.); mwu01@stanford.edu (M.W.)

**Keywords:** microfluidics, microbubble generation, bubble size control, cavitation nuclei

## Abstract

Microbubbles, acting as cavitation nuclei, undergo cycles of expansion, contraction, and collapse. This collapse generates shockwaves, alters local shear forces, and increases local temperature. Cavitation causes severe changes in pressure and temperature, resulting in surface erosion. Shockwaves strip material from surfaces, forming pits and cracks. Prolonged cavitation reduces the mechanical strength and fatigue life of materials, potentially leading to failure. Controlling bubble size and generating monodispersed bubbles is crucial for accurately modeling cavitation phenomena. In this work, we generate monodispersed microbubbles with controllable size using a novel and low-cost microfluidic method. We created an innovative T-junction structure that controls the two-phase flow for tiny, monodispersed bubble generation. Monodisperse microbubbles with diameters below one-fifth of the channel width (W = 100 µm) are produced due to the controlled pressure gradient. This microstructure, fabricated by a CNC milling technique, produces 20 μm bubbles without requiring high-resolution equipment and cleanroom environments. Bubble size is controlled with gas and liquid pressure ratio and microgeometry. This microbubble generation method provides a controllable and reproducible way for cavitation research.

## 1. Introduction

### 1.1. Applications of Microbubbles

Microbubble generation is widely applied in the fields of biomedicine, environmental science, the food industry, and experimental modeling of cavitation, etc. In the biomedical field, microbubbles serve not only as efficient drug delivery tools but also as functionalized carriers for various therapeutic molecules such as drugs and genes [1]. Additionally, microbubbles can also be used as tools for gas embolism, effectively killing cancer cells by blocking the capillaries around tumors and cutting off the supply of oxygen and nutrients [2,3]. Microbubbles also enhance ultrasound imaging, improving the clarity and detail of scans, which increases the accuracy of diagnoses and treatments [4,5]. In environmental applications, microbubbles play a crucial role in wastewater treatment. For example, the strong oxidizing properties of the ozone microbubbles provide a highly efficient way for a range of purification processes, including the sterilization and removal of colors, odors, and organic pollutants in both water purification and sewage treatment [6]. In the food industry, microbubbles play diverse roles, including cleaning, disinfection, gas transport, enhancing food material properties, and mimicking mouthfeel, offering new technological solutions for food processing and production [7].

Microbubbles also play a crucial role in the study of cavitation erosion. These bubbles, acting as cavitation nuclei, experience a cycle of expansion, contraction, and eventual collapse. The collapse of these bubbles generates shockwaves, alters local shear forces, and leads to an increase in local temperature [8]. Cavitation in fluid systems triggers severe local changes in pressure and temperature, resulting in surface erosion. The shockwaves produced can strip material from the surface, forming pits and cracks [9,10]. Prolonged exposure to cavitation gradually reduces the mechanical strength and fatigue life of materials, potentially leading to failure. Moreover, in environments like ships and pump systems, bubble cavitation not only accelerates material erosion but also creates substantial noise and vibrations [10]. Since these phenomena can cause significant material erosion, it is important to model bubble cavitation and research bubble flow physics to deepen our understanding of cavitation erosion and develop effective solutions.

### 1.2. Microbubble Generation Technology

There are several methods to generate microbubbles, each offering specific benefits and limitations. Sonication-based microbubble generation methods utilize high-intensity ultrasound waves to disperse gases or liquids into a suspension containing a capping material to form microdroplets/microbubbles with adsorbed proteins or surfactants on the surface [11]. However, this method produces bubbles with wide size distribution, requiring filtration to remove oversized bubbles and mitigate embolization risks. In addition, the size of microbubbles depends on the frequency, power, and pulse regime, but there is a lack of clear theoretical models to precisely control the relationship between the ultrasound parameters and the size distribution of microbubbles [12]. Another widely used method involves using high shear mixing to emulsify a polymer dissolved in a suitable solvent into an aqueous suspension [13]. As the solvent evaporates, the polymer forms a shell around the droplets, resulting in microbubbles. The size distribution of the microbubbles depends on initial emulsified droplets and potential fragmentation or merging during processing, often requiring additional steps to meet the stringent size requirements [14]. Membrane emulsification technology produces emulsions by repeatedly forcing components through a porous membrane, producing droplets that can either be processed into microbubbles or directly form gas microbubbles [15]. This method offers precise size control, relying on membrane properties such as pore size distribution, hardness, wettability, and surface treatment [16]. Inkjet printing enhances microbubble uniformity by injecting fluid through a stainless-steel nozzle using a pressure pulse generated by a piezoelectric crystal. One key benefit is the ability to adjust droplet size by modifying the frequency and duration of the pressure pulses [17]. While the technique produces relatively uniform bubbles, the achievable size range is limited by the nozzle size, and the small nozzle size is prone to clogging. Recently, microfluidic techniques have been applied to generate more uniform bubbles with precise control. There are three main types of microfluidic devices to generate microbubbles: co-flow, flow-focusing, and crossflow. Microfluidic devices have several advantages. These devices allow for the precise regulation of bubble size, reduce gas and liquid consumption. They are also scalable for higher production throughput via parallelized designs. Additionally, their flexible structural configurations enable the generation of microbubbles with diverse sizes, shapes, and structures, making them suitable for various applications [18,19].

### 1.3. Principle of Microbubble Generation

The design of efficient and low-cost bubble generation devices is important for various applications. Microbubbles are typically generated using three methods [20]. The first method is to compress air into liquid and release it through a specially designed nozzle system to form small bubbles based on the cavitation principle [21,22]. The second method induces bubble cavitation by applying high-power ultrasound at points of extreme rarefaction in standing ultrasonic waves [23]. The third method utilizes low-offset pressure air flow, integrated with additional mechanisms, such as mechanical vibration, flow focusing, or fluidic oscillation, to break up bubbles [24]. The first two-phase tip enters the liquid channel, and the gaseous protrusion elongates in the liquid channel. Under the shear force of the liquid and the resultant pressure gradient, it is broken into bubbles, the process often referred to as “pinch off” [19]. There are several factors affecting bubble generation, including the fluid physical properties [25], microchannel geometries [26], the velocity or pressure ratio of the two-phase fluids (gas and liquid), etc. [27]. These factors impact the size, stability, uniformity, and generation rate of the bubbles [28,29].

Among the bubble generation microfluidic devices, T-/Y-shaped microstructures have been widely studied and applied to various applications, attributed to their simplicity and versatility. Although the T-junction structure for bubble generation is simple, it is difficult for a T-junction microfluidic device to produce very small bubbles because the geometry limits the “pinch-off” process. In other words, in a T-junction, the bubble is “squeezed” off at the intersection, and the final size is limited by the width of the channel where the gas phase enters [30]. The conventional T-junction method can generate bubbles ranging from 50 to 500 μm, which limits the application of bubbles for cavitation studies, ultrasound imaging, and drug delivery. To control the bubbles’ size and uniformity, advancements such as the capillary-embedded T-junction microfluidic device have been developed. This design enables the production of microbubbles with more precise sizes ranging from 65 to 200 μm [31,32]. Additionally, modifications to the geometry of T-junctions, such as incorporating Y-shaped structures, have been investigated. Studies on these configurations show that altering the angle between channels can significantly influence bubble size from 800 to 3100 μm [33]. Additionally, parallel double T-junction designs have been introduced, enabling the production of bubbles ranging from 200 to 500 μm [34].

Producing small bubbles requires microchannel features of comparable dimensions [35], which can reduce the reliability of the continuous bubble generation due to the clogging issue and increase the fabrication cost. To overcome these challenges, in this work, we used a novel T-junction structure capable of producing monodispersed bubbles that are smaller than one-fifth the size of the microchannel. This method eliminates the need for complex microfabrication processes, thereby improving reliability and reducing production costs. Our innovation lies in introducing a specific geometrical microstructure at the junction of the two fluid channels, which plays an important role in regulating the flow and generating monodisperse bubbles with a size as small as 20 µm. This specific geometry provides an effective method to generate monodisperse microbubbles in a more stable and consistent manner, laying the foundation for subsequent practical applications.

### 1.4. Fabrication Methods for Microfluidic Bubble Generation Devices

There are various manufacturing methods to create the necessary structures for the microbubble generator, including photolithography [36], micromilling [37], 3D printing [38], and laser ablation [39]. Photolithography uses UV light and photosensitive materials to create precise microstructures with a resolution of ~1–2 µm. The advantages of photolithography include high precision and good repeatability, enabling the creation of complex structures. However, the fabrication procedure is complex and requires a cleanroom environment. Compared to photolithography, CNC micromilling is cost-effective and suitable for mass production using mechanical milling techniques. However, the precision of CNC micromilling to fabricate the microstructures with high resolution is limited (~30–50 µm). The 3D printing technology is flexible in design and suitable for rapid prototyping of complex geometric structures [38]. Yet, its production precision and surface smoothness are lower than those achieved with CNC machining and photolithography. Laser ablation can etch structures on the material surface, offering high production efficiency [36], but it also faces challenges in precision and depth control.

In this study, we developed a microbubble generator that does not require high-resolution fabrication techniques. Using micro-milling machine technology, we successfully generated bubbles with sizes down to 20 μm without the need for a high-resolution lithography process. Our method provides a cost-effective and simple way compared to conventional photolithography. Producing a single photolithography-fabricated device can cost about USD50–USD100 when factoring in materials and cleanroom usage. These costs are compounded for iterative testing of different geometries, as each new design requires a new mask and significant setup time [40,41]. In contrast, our micromilling approach eliminates the need for cleanroom facilities and expensive photolithography equipment. We utilize acrylic plates (USD5–USD10 per sheet) and milling bits (~USD10–USD50 each) to fabricate the mold, making per-device costs approximately USD1–USD5. Furthermore, the flexibility of micromilling enables rapid prototyping and fabrication of multiple designs without the additional cost of new masks, drastically reducing the cost of iterative development.

## 2. Material and Methods

### 2.1. Device Design and Fabrication

**Figure 1:**
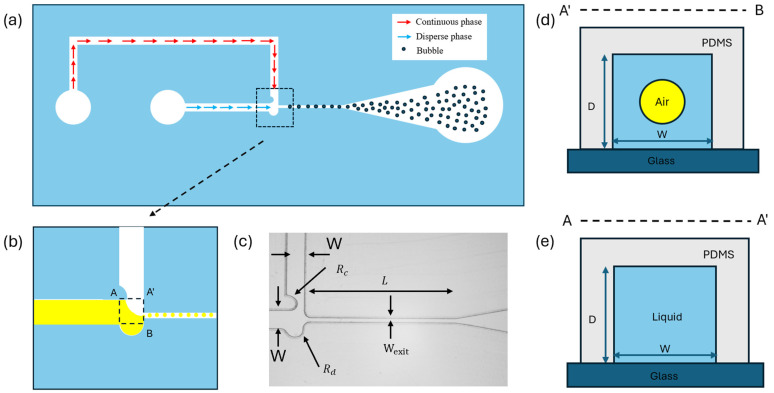
Schematic illustration and mechanism of the microbubble generator. (**a**) Working mechanism of microbubble generation. (**b**) A zoomed-in view of the T-junction where the two phases meet. The gas flows (dispersed phase) horizontally (yellow), while the liquid flows (continuous phase) vertically (white), leading to bubble formation downstream. (**c**) Schematic illustration of the T-junction microfluidic structure. (**d**) Schematic illustration showing a cross-section of a microfluidic channel upstream. (**e**) Schematic illustration showing a cross-section of a microfluidic channel downstream.

The schematic of the microfluidic microbubble generation device and the process of microbubble generation are shown in Figure 1a. The cross-sectional area of bubble generation is shown in Figure 1d,e. The device has two inlets and one outlet, including one inlet for gas injection, one inlet for liquid injection, and one outlet for bubble collection. Microbubbles are generated through a T-junction structure with two semi-circular shapes, where the gas and liquid meet orthogonally. As shown in Figure 1c, the semi-circular structure at the end of the dispersed phase has a radius (R_d_) of 100 μm, and the semi-circle in the continuous phase has a radius (R_c_) of 150 μm. The height (D) of the microbubble generation device, the width (W_exit_), and the length (L) of the exit channel are W_exit_=50 μm, 50 μm, and L = 1600 μm, respectively. The widths of the channel where the gas and liquid phase enters (W) are both set at 200 μm. In Section 3.4, we changed the depth of the microbubble generation device and the widths of the channel where the gas and liquid enter (W) to investigate how these geometric dimensions affect the size of the bubble.

To fabricate a microfluidic microbubble generation device, micromilling and soft lithography techniques were employed. The geometric shapes and patterns of the microfluidic structures were designed using Creo Parametric 10.0 software (PTC, Boston, MA, USA). The designed geometric structures were then fabricated on a polymethyl methacrylate (PMMA) board by a CNC micromilling machine (Minitech Machinery Corp., Norcross, GA, USA). Next, polydimethylsiloxane (PDMS, Sylgard 184, Dow Corning, Midland, MI, USA) was used as the material to replicate the geometric structures on the mold. The PDMS prepolymer and curing agent were mixed at a 10:1 ratio, and a vacuum pump was used to remove microbubbles from the mixture, preventing air pockets from entering the structures and interfering with subsequent experimental observations. After 20 min under vacuum, the PDMS was poured onto the mold surface and then cured in an oven at 65 °C. Once cured, the PDMS layer was carefully peeled from the mold, and holes were punched for the inlet and outlet ports. The PDMS layer was then irreversibly bonded to a glass slide using plasma treatment (Electro-Technic Products, Chicago, IL, USA). The assembled microfluidic device was placed back in the oven and heated at 65 °C for 5 min, then left to sit for 24 h to ensure the PDMS was fully cured and bonded with the glass. Before using the microfluidic device, SDS solution was injected into the microfluidic chip and left for 5 min to fully wet the channels, ensuring the internal hydrophobicity of the channels.

### 2.2. Experimental Setup

**Figure 2:**
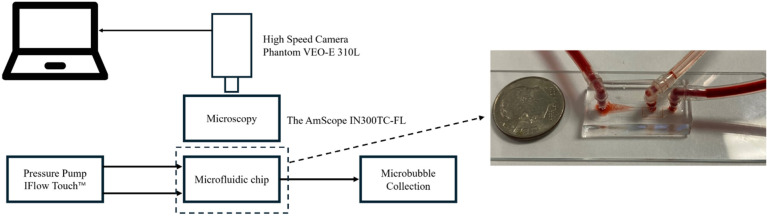
Schematic illustration of the experimental setup and an optical image of the microfluidic microbubble generator.

The experimental setup is shown in Figure 2. In the experiments, a pressure pump (iFlow Touch™ Microfluidic Pump System Precigenome, San Jose, CA, USA) was used to inject air and liquid. We chose this pressure pump because it could maintain stable air pressure with minimal fluctuation, which is important for producing stable and uniform bubbles. A 5% SDS (Sodium Dodecyl Sulfate) solution was used as the liquid medium, which helps in wetting surfaces to facilitate bubble formation and increase their stability. A microscope (United Scope LLC, Irvine, CA, USA) and a high-speed camera (Vision Research, Wayne, NJ, USA) were used to observe the bubble formation process (Videos S1 and S2).

### 2.3. Data Analysis

We used ImageJ 1.54 to process and analyze the microscopic images. First, the raw microscope images were imported into the software and calibrated for pixel size using a standard scale to ensure that subsequent measurements were recorded in microns (µm). The image was then converted to an 8-bit grayscale to simplify the information and reduce color noise. Next, using Gaussian filtering, the image was smoothed, and the bubble boundaries were highlighted. To clearly separate the bubbles from the background, we applied Otsu thresholding to binarize the image and ensure that the bubbles are clearly visible by fine-tuning the brightness and contrast where necessary. After thresholding is applied, the bubbles are displayed as white particles on a black background in the binarized image. After obtaining the binary image, we used the “Analyze” function to automatically recognize and measure bubble features. Based on a pre-existing range of bubble sizes, we set appropriate area and roundness parameters to exclude noise and irregular particles. By selecting parameters such as “Area” and “Perimeter”, we obtain statistical data on the bubbles. These results provide the basis for subsequent characterization of bubble distribution uniformity and size.

## 3. Results and Discussion

### 3.1. Working Mechanism of the Microbubble Generation

During the bubble generation process in this T-junction structure, a microbubble experiences various external forces. To describe the bubble’s behavior of detachment, an equivalent spherical model for bubble growth was developed using the Runge–Kutta numerical method based on the force balance relationship [42,43]. A theoretical model was developed based on the fundamental assumptions of ellipsoidal bubble shapes. The analysis indicates that during the phases of bubble growth and detachment, microbubbles are primarily influenced by surface tension, gas thrust, liquid drag, and inertial forces [44]. This device uses a T-junction structure to produce microbubbles, following the principles of generation as outlined in the formulas [43].

(1) $$F_{\sigma }=\pi \cdot d_{0}\cdot \sigma$$

σ is the surface tension coefficient. *d*_0_ is the equivalent diameter of the stoma and its calculation formular is

(2) $$d_{0}=\frac{\sqrt{4\;\cdot \;l\;\cdot \;h}}{\pi }$$

where l is the gas channel width, and h is the height of the microfluidic channel [43].

The liquid pulling force ($F_{D}$) is the force generated by the interaction of the liquid flow inside the channel and the liquid viscosity, where $v_{eff}$ is the relative velocity of the bubble and liquid phase; $C_{D}$ is the liquid pulling force coefficient; $\rho_{l}$ is the liquid density [43].

(3) $$F_{D}=\frac{\pi }{4}\cdot d_{s}^{2}\cdot C_{D}\cdot \frac{1}{2}\cdot \rho_{l}\cdot v_{eff}^{2}$$

Gas thrust ($F_{M}$) is the force that the gas which flows into the bubble at a certain speed pushes the bubble interface, where $v_{g}$ is the gas flow velocity, and $\rho_{g}$ is the gas density [43].

(4) $$F_{M}=\frac{\pi }{4}\cdot d_{0}^{2}\cdot C_{D}\cdot \rho_{g}\cdot v_{g}^{2}$$

(5) $$F_{i}=\frac{d}{d_{t}}[\rho_{g}\cdot V_{B}\cdot \frac{dS}{dt}+C_{MC}\cdot V_{B}\cdot \rho_{l}\cdot (\frac{dS}{dt}-v_{l}i)]$$

Inertial force ($F_{i}$) is the force the inertia exerts on the gas–liquid interface, keeping the bubble in its original state of motion, where $V_{B}$ is the volume of the bubble; S is the bubble center point displacement, and $C_{MC}$ is an accessional quality coefficient. The bubble is generated by a T-junction structure in our design, which follows the principles of generation as outlined in these formulas [43].

### 3.2. Effect of Geometry of T-Junction Structure on Bubble Size

We designed four geometries of T-junction microfluidic structures to investigate how semi-circular elements influenced bubble generation. The first structure is a standard T-junction without any added elements. The second structure includes a semi-circular element at the end of the liquid channel. The third structure has a semi-circular element at the end of the gas channel. The fourth structure features two semi-circular elements positioned at the intersection of the gas channel (disperse phase) and the liquid channel (continuous phase). By comparing these four configurations, we aim to understand the specific effects of semi-circular elements in each channel on bubble formation and how their combined presence impacts bubble characteristics.

**Figure 3:**
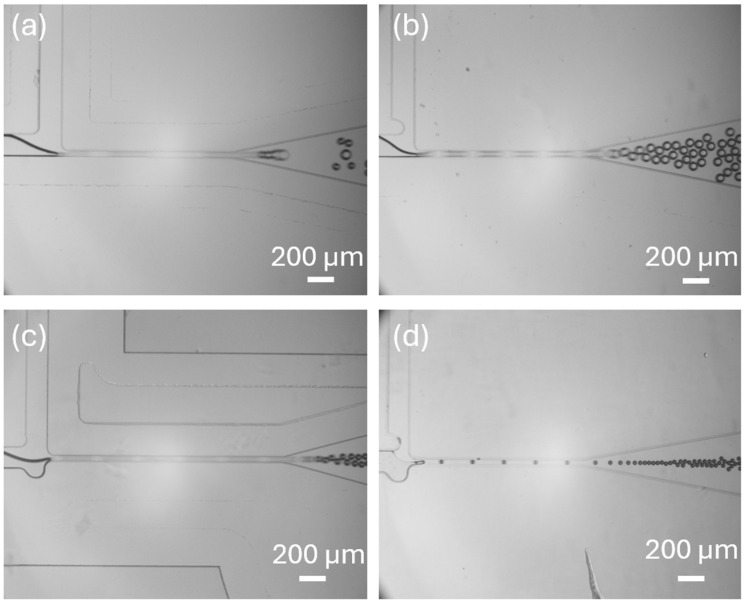
Bubble generation using four microbubble generators with four different T-junction geometries. (**a**) Standard T-junction structure. (**b**) T-junction structure with a tip. (**c**) T-junction structure with a cavity. (**d**) T-junction structure with a cavity and a tip.

During this experiment, all structures were tested for bubble generation under the same pressure conditions with a gas pressure of 200 mbar and a liquid pressure of 300 mbar, allowing for the direct comparison of bubble generation across the different designs. The experimental results, as shown in Figure 3 and Videos S3–S6, show distinct differences in bubble generation. The T-junction structure without a semi-circular element generates ununiform size and the largest bubbles among all structures (Figure 3a). The semi-circular structure at the end of the liquid channel and gas channel has a narrower gas–liquid interface (Figure 3b,c). The fourth structure, a T-junction structure with both a tip and a cavity, exhibits the narrowest gas–liquid interface, generating the smallest bubbles and most consistent bubble among the structures tested (Figure 3d).

**Figure 4:**
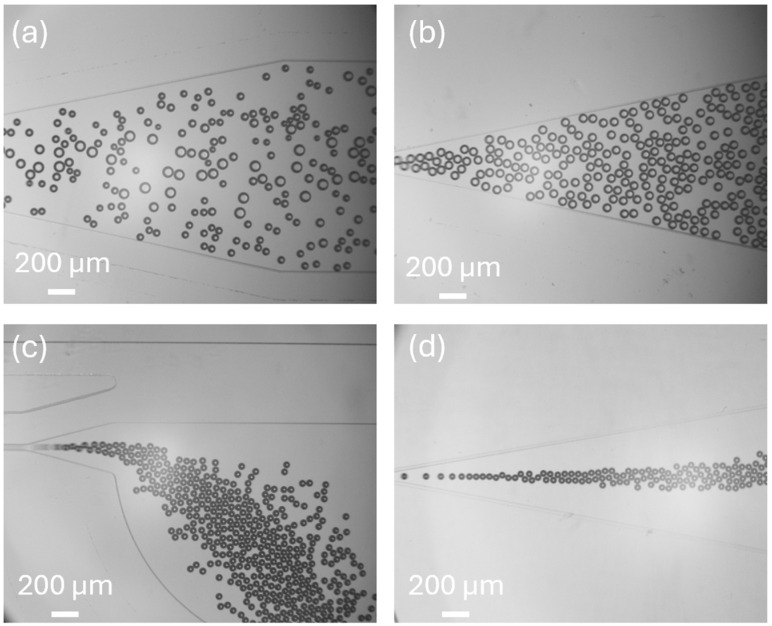
Bubbles in collection chambers. (**a**) Bubbles generated by the device with standard T-junction structure. (**b**) Bubbles generated by the device with T-junction structure that includes a tip. (**c**) Bubbles generated by the device with the T-junction structure that includes a cavity. (**d**) Bubble generated by the device with T-junction structure that includes a cavity and a tip.

**Figure 5:**
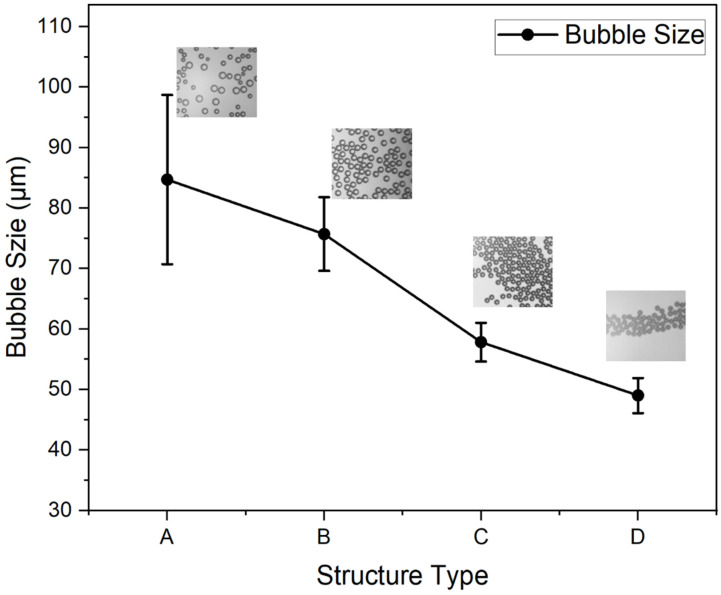
Bubble size generated by the four devices with different types of structure. A: Standard T-junction structure. B: T-junction structure with a tip. C: T-junction structure with a cavity. D: T-junction structure with both a tip and a cavity.

Figure 4 shows the microbubbles collected by devices with different T-junction structure geometries. The average size of bubbles generated by different devices is shown in Figure 5. The microbubble generation device with the standard T-junction structure produced bubbles with an average size of 84.7 μm (±14.6 μm). In comparison, the structure with a tip produced bubbles with an average size of 75.6 μm (±6.1 μm), and the structure with a cavity produced smaller bubbles, measuring 57.8 μm (±3.2 μm). The combination of both tip and cavity structures resulted in the smallest bubbles, with an average size of 48.9 μm (±2.9 μm). These findings demonstrate that structures with a tip and cavity generate smaller and more uniform bubbles compared to the standard T-junction structure. The T-junction structure with the cavity contributes more significantly to bubble size reduction than the structure with the tip. Due to the considerable impact of combining both the tip and cavity structure in minimizing bubble size, we select the device with this configuration for further analysis and optimization.

### 3.3. Effect of Gas-to-Liquid Pressure Ratio on the Bubble Size

The purpose of studying the effect of gas-to-liquid pressure ratio on bubble size in a T-junction structure with both a tip and a cavity is to evaluate if adjusting the gas-to-liquid pressure ratio can effectively control bubble size. We aim to establish optimized gas-to-liquid pressure ratios that can control the bubble size to be used for cavitation research.

**Figure 6:**
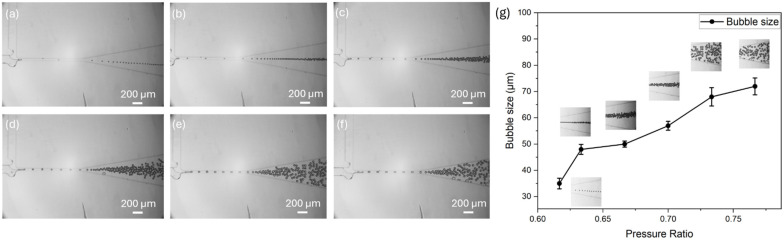
Bubble generation process at different gas-to-liquid pressure ratios: (**a**) 185 mbar gas/300 mbar liquid; (**b**) 185 mbar gas/300 mbar liquid; (**c**) 200 mbar gas/300 mbar liquid; (**d**) 210 mbar gas/300 mbar liquid; (**e**) 220 mbar gas/300 mbar liquid; (**f**) 230 mbar gas/300 mbar liquid; (**g**) bubble size at different pressure ratios.

In this experiment, we tested six different gas-to-liquid pressure ratios using the same T-junction structure with both a tip and a cavity. To ensure the consistency, we keep the liquid pressure constant at 300 mbar with varying gas pressure: (a) 185 mbar; (b) 190 mbar; (c) 200 mbar; (d) 210 mbar; (e) 220 mbar; and (f) 230 mbar. The results, as shown in Figure 6g, indicate a positive correlation between bubble size and pressure ratio. This shows that generating smaller bubbles requires a lower pressure ratio. Therefore, to produce smaller and more uniform bubbles within this structure, we can operate at a lower gas-to-liquid pressure ratio by either decreasing the gas pressure or increasing the liquid pressure. By adjusting the pressure ratio, we can achieve more precise control over bubble size in this structure.

### 3.4. Effect of Channel Geometry on Bubble Size Under Different Pressures

To further optimize the device for precise control of the bubble size, we evaluated the effect of channel depth on bubble size over a range of applied pressures. Bubble generation was tested using the microbubble generation devices with the depth of 50 μm and 100 μm. During this experiment, the gas-to-liquid pressure ratio (2:3) and inlet channel geometry (W = 200 μm) remained constant to ensure controlled conditions. We systematically applied a wide range of gas and liquid pressures from 50 mbar to 250 mbar in devices with different depths, allowing us to assess the effect of the depth of the channel on the bubble size under a wide range of pressures.

The results show a decrease in bubble size as the pressure incrementally increased for both devices. With increasing liquid pressure from 75 mbar to 375 mbar, the bubble size decreases. The decrease in bubble size was more pronounced in the lower pressure range (75 mbar to 150 mbar pressure), while the reduction became less significant at higher pressures (187.5 mbar to 375 mbar). For the device with a 50 µm channel depth, larger bubbles were generated at lower pressures (50 mbar to 150 mbar) and had a significant reduction in size when the liquid pressure reached 150 mbar. This illustrates the critical role of pressure in this range in regulating bubble size. The device with a 100 µm channel depth followed a similar trend. This device generated bubbles with smaller sizes at a lower pressure range (from 75 mbar to 150 mbar). However, at higher pressure, the bubble sizes align with those generated in the 50 µm depth channel.

**Figure 7:**
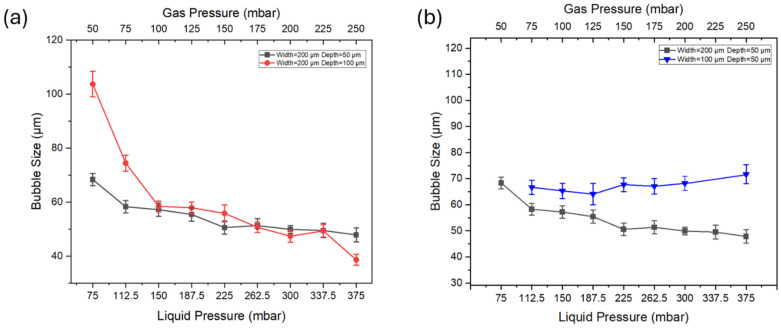
(**a**) Bubble size under different pressures with different channel depths. (**b**) Bubble size under different pressures with different channel widths.

In addition to investigating the channel depth on the bubble generation, we also explored the effect of channel width on bubble formation. Bubble generation is evaluated by comparing bubble generation devices with the widths of the channel (W) of 100 μm and 200 μm. As shown in Figure 7b, for the device with a channel width of 100 μm, there is no significant change in bubble size with an increase in gas and liquid pressure. For the device with a channel width of 200 μm, the bubble size gradually decreases with increasing pressure. The experimental results show that 200 μm channels allow for more precisely controlling bubble size, especially compared to the device with a channel width of 100 μm, where the decrease in bubble size is less significant and less predictable under the same conditions.

We also found that bubbles formed in the channel with a width of 100 μm were larger than those in the 200 μm channel across the entire pressure range. This difference in bubble size may relate to the dynamic behavior of bubbles within the narrow channel. Specifically, bubbles in the 100 μm channel quickly expand to or exceed the channel width after formation, thereby being compressed by the channel walls, affecting their final size and stability. Additionally, in the device with a channel width of 100 μm, microbubbles could not be generated at liquid pressure of 75 mbar and 337.5 mbar. These findings indicate that narrower channels may have limitations on stable bubble formation.

### 3.5. Effect of Channel Geometry on Bubble Homogeneity

Uniformity is an important factor for applications requiring consistent bubble size, as variations in size can affect performance and stability. To evaluate the uniformity of bubbles, we introduce the polydispersity index (PDI). PDI is a commonly used metric to assess the uniformity of a distribution, defined as the ratio of the weight-average molecular weight to the number-average molecular weight [44]. In microfluidic applications, a PDI of less than 5% is considered a standard for excellent bubble uniformity, indicating that the bubble sizes produced are highly consistent [31,45]. The PDI (σ) for describing the bubble size distribution can be expressed using Equation (6):

(6) $$\sigma =\frac{\delta }{D}\times 100\%$$

where D is the average equatorial diameter (D); δ is the standard deviation of measured equatorial diameters [46].

**Figure 8:**
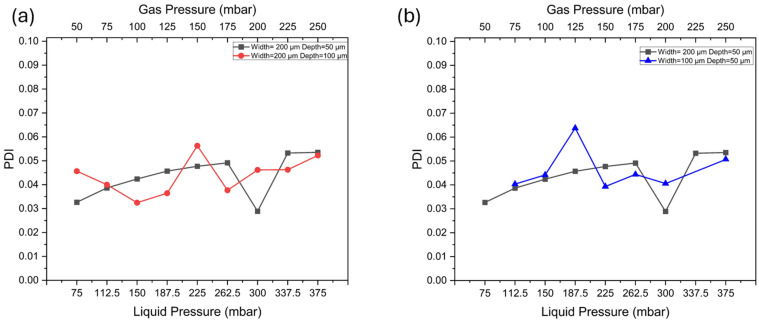
(**a**) Bubble PDI in the devices with different channel depth (D). (**b**) Bubble PDI in the devices with different channel widths (W).

We examined the uniformity of bubbles produced in the device with different depths and widths to investigate if the channel geometry can affect bubble uniformity. According to Figure 8a, the generated bubbles demonstrated a good performance in uniformity. In the device with a channel depth of 50 μm, the PDI does not exceed 5% among all the tested pressure ranges. Compared with the device with a depth of 100 μm, the PDI has fewer fluctuations. This indicates that the device with a channel depth of 50 μm performs better in producing uniform bubbles under varying pressures. We also compare the bubble uniformity in the device with different channel widths. As shown in Figure 8b, for the device with a width of 100 μm, the PDI fluctuations were higher, indicating greater instability in narrower channels across the pressure range. This instability in bubble formation under varying pressure could present challenges for applications that require precise size control by adjusting injection pressure.

To create cavitation nuclei with the desired size and uniformity for cavitation research, we conducted experiments to optimize the geometry of the bubble generation device. We compared the optimal bubble size and uniformity of the three devices with different channel geometries. The three devices are (1) the device with a channel width of 200 μm and a depth of 100 μm, (2) the device with a channel width of 200 μm and a depth of 50 μm, and (3) the device with a channel width of 100 μm and a depth of 50 μm. These devices were evaluated based on the average bubble size and polydispersity index (PDI) to determine which geometry produced the desired cavitation nuclei.

**Figure 9:**
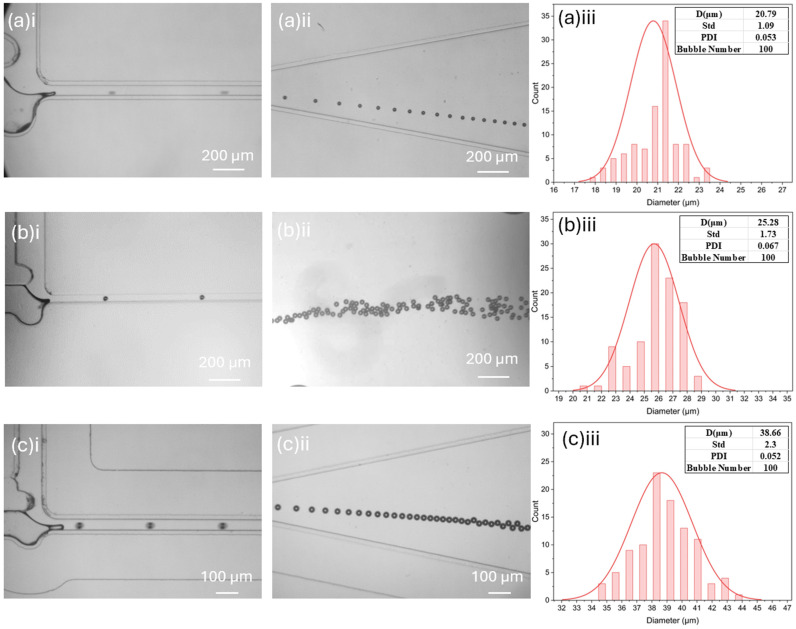
(**a**) Minimum-sized bubbles generated in a T-junction with D = 100 μm; W = 200 μm. i. Microbubble generation. ii. Microbubbles collected in the chamber. iii. Microbubble size distribution and polydispersity. (**b**) Minimum-sized bubble generated in a T-junction with D = 50 μm; W = 200 μm. i. Microbubble generation. ii. Microbubbles collected in the chamber iii. Microbubble size and polydispersity. (**c**) Minimum sized bubble generated in a T-junction D = 50 μm; W = 100 μm. i. Microbubble generation. ii. Microbubbles collected in the chamber. iii. Microbubble size and polydispersity.

The bubble generation process of the three devices is shown in Figure 9. For each device, 100 bubbles were measured to ensure a reliable assessment of bubble size and uniformity. As shown in Figure 9a, the structure with a channel width of 200 μm and depth of 100 μm produced bubbles with an average size of 38.66 μm and a polydispersity index (PDI) of 0.05251 (5.251%). The device with a width of 200 μm and a depth of 50 μm generated bubbles with an average size of 20.79 μm and a PDI of 0.05281 (5.28%), meeting the requirements for our cavitation erosion experiments due to the small size and low PDI of the bubbles. In Figure 9c, the device with a channel width of 100 μm and a depth of 100 μm produced bubbles with an average size of 25.68 μm and a higher PDI of 0.0675 (6.75%). Given the performance of the three devices, we selected the device with a width of 200 μm and a depth of 50 μm for further experiments, as it consistently produced the smallest and most uniform bubbles. This device is capable of generating 20 μm monodispersed bubbles, making it suitable for use as cavitation nuclei in a broad range of cavitation research applications.

### 3.6. Effect of Time on Bubble Size

**Figure 10:**
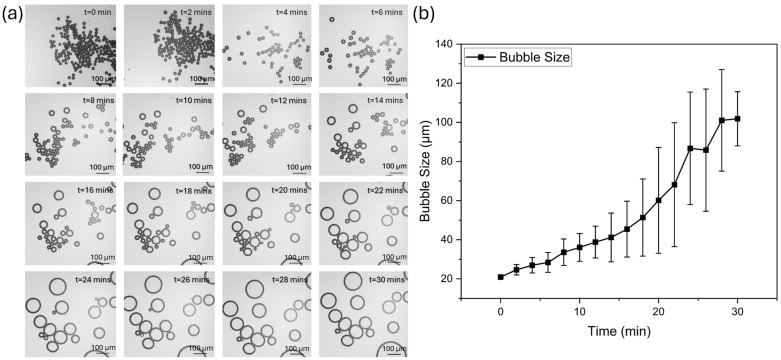
Bubble size evolution over time. (**a**) Sequential images showing bubble size evolution over time at different time points (0–30 min). Initially, the bubbles are small and uniform in size. As time progresses, the bubbles undergo cycles of collapse and expansion, leading to an overall increase in size. (**b**) Quantitative analysis of bubble size evolution over time. Bubble size continuously increased throughout the observation period.

To evaluate the suitability of bubbles for cavitation testing, it is important to assess the stability of the bubbles over time. In this section, we aimed to test the stability of 20 μm bubbles generated by the microbubble generation device with an optimized T-junction structure. For this purpose, the generated bubbles were collected at the end of the microfluidic chip and photographed every 2 min under a microscope, with the entire observation lasting 30 min (Figure 10a). The average size is measured from 0 to 30 min at 2-min intervals (Figure 10b). These results indicate that the bubble size does not have a significant change and remains relatively uniform within the first 6 min. After this period, the bubbles begin to undergo both collapse and expansion, which is due to the change in the pressure inside the bubble and the pressure in the liquid phase [47]. The results showed that the bubble size continuously increased throughout the observation period. It is noted that the isolated bubble has greater size variations compared to those in contact with each other, indicating that clustered bubbles experience less size fluctuation. Additionally, the observed size increase in isolated bubbles can be attributed to gas diffusion effects under Laplace pressure. Specifically, the internal Laplace pressure in smaller isolated bubbles is higher due to their increased curvature, causing a diffusion-driven transfer of gas from the interior of the bubble to the surrounding liquid phase. This gradual influx of gas contributes to bubble growth over time [48].

## 4. Conclusions

To conclude, we successfully developed a low-cost microbubble generation device for producing monodispersed bubbles in a highly controllable way, avoiding using the complex photolithography process that is commonly used for microfluidic bubble generators. Through this research, we designed a T-junction structure with two semi-spherical elements (tip and cavity) that can regulate the liquid flow and gas flow to stabilize and control the microbubble size. Also, we experimentally investigated and optimized the parameter of the device geometry to produce the microbubble size down to 20 μm with high uniformity. Due to its high controllability, small size, and uniformity, the microbubble generation device provides an effective method for generating cavitation nuclei, enabling accurate modeling in cavitation research.

## Data Availability

The original contributions presented in this study are included in the article/Supplementary Materials. Further inquiries can be directed at the corresponding author.

## Supplementary Materials

The following supporting information can be downloaded at https://www.mdpi.com/article/10.3390/mi15121531/s1, Video S1: Microbubble generation; Video S2: Microbubble generation-2; Video S3: Standard T-Junction structure; Video S4: T-Junction structure with a cavity; Video S5: T-Junction structure with a tip; Video S6: T-Junction structure with a tip and a cavity.
